# Early (<6 Days) Extracranial Carotid Revascularization After Intravenous Thrombolysis for Stroke: A Scoping Review

**DOI:** 10.3390/jcm15031174

**Published:** 2026-02-02

**Authors:** Giovanni Coppi, Luiz Felippe Milazzo, Marco Damiano Pipitone, Giovanni Zambello, Francesco Zaraca, Reinhold Perkmann

**Affiliations:** Vascular and Thoracic Surgey Unit, Central Hospital of Bolzano, 39100 Bolzano, Italy; luizfelippe.milazzo@sabes.it (L.F.M.); marcodamiano.pipitone@sabes.it (M.D.P.); giovanni.zambello@sabes.it (G.Z.); francesco.zaraca@sabes.it (F.Z.); reinhold.perkmann@sabes.it (R.P.)

**Keywords:** scoping review, intravenous thrombolysis, carotid endarterectomy, carotid artery stenting, intracranial hemorrhage

## Abstract

**Background**: Current guidelines recommend delaying extracranial carotid revascularization for at least 6–7 days after intravenous thrombolysis (IVT). However, evidence remains inconclusive, and patients with minimal or no brain lesions may benefit from earlier intervention. **Objective**: This scoping review evaluates outcomes of carotid revascularization performed within six days of IVT in patients with small strokes or no imaging-detected lesions. **Design**: We searched Medline, EMBASE, and Cochrane CENTRAL for studies published between 2005 and 2025 reporting carotid revascularization after IVT. Primary outcomes included perioperative ischemic stroke, symptomatic intracranial hemorrhage (sICH), asymptomatic intracranial bleeding (aICB), and wound complications. Data on timing, imaging findings (CT/MRI), and stroke severity (NIHSS or modified Rankin scale) were extracted. The review followed PRISMA-ScR guidelines. **Results**: Seventeen studies (1459 patients) were included; 97.6% underwent carotid endarterectomy (CEA) and 2.4% carotid artery stenting (CAS). Data for procedures within six days post-IVT were available for 402 patients, with some treated within 24–72 h. Mean NIHSS at admission was 10.2 ± 2.8. Brain lesion characterization was poor and only used as an exclusion criterion. Thirty-day ischemic stroke incidence was 2.5%, while combined sICH/aICB was 4.0%, with symptomatic cases at 2.5%. Most hemorrhages occurred in patients operated within 48–72 h from IVT and in patients with higher NIHSS. CAS carried higher sICH risk than CEA (8.6% vs. 1.9%). Overall mortality was 1.1%; wound complications occurred in 3.7%. **Conclusions**: The available literature provides limited characterization of brain lesion extent. Neurological complications after CEA are rare, mainly limited to patients operated within 48–72 h from IVT. Further research is needed to optimize treatment in this patient group, although delaying CEA only to 48–72 h seems reasonable in case of low NIHSS.

## 1. Introduction

Today, intravenous thrombolysis with rTPA (IVT) is the mainstay of ischemic stroke treatment if the patient presents within 4.5 h from symptoms onset [1]. Its use has faced a steady increase in the last 20 years, reaching almost 10% of all stroke admissions [2].

Extracranial internal carotid artery (ICA) stenosis accounts for approximately 10–20% of ischemic strokes. Current evidence strongly supports revascularization of 50–99% symptomatic stenoses in appropriately selected patients within 14 days of symptom onset, as this timing is critical to reduce the risk of stroke recurrence [3].

The extensive implementation of IVT has thus resulted in a progressive rise in the incidence of symptomatic ICA stenoses requiring subsequent intervention, thereby underscoring the critical need to evaluate the safety of revascularization in this specific setting and to define the optimal timing [4].

Revascularization should, in fact, be timed carefully: performing it too early may increase bleeding risks associated with IVT, such as intracranial hemorrhage (ICH) and surgical site bleeding. Conversely, delaying the procedure too long can raise the likelihood of recurrent stroke, which may be up to 5.5–22% in the first week in patients with symptomatic ICA stenosis [5,6,7,8,9].

The latest guidelines from the European Society for Vascular and Endovascular Surgery recommend postponing carotid revascularization for approximately six days following IVT, due to an increased risk of intracerebral hemorrhage and cervical hematoma. This carries a Class IIa recommendation with Level of Evidence B [3], primarily based on a meta-analysis by Kakkos et al. [5].

It is still uncertain whether patients with minimal or no cerebral ischemic involvement, who naturally present a lower risk of ICH [10], can safely undergo earlier revascularization following thrombolysis, thereby potentially decreasing the likelihood of recurrent stroke and shortening hospital stay [3].

The aim of this scoping review is to therefore systematically analyze the published results of carotid revascularization after thrombolysis to see if early revascularization after thrombolysis can be safely performed in patients with small or no brain lesions. Severity of stroke is used as a surrogate marker for the extension of the lesion in case a definite report on the dimension of the lesion cannot be identified [11,12]. The data produced will be used to assess if further studies on the topic are needed and if a multicenter dedicated randomized controlled trial could be run.

## 2. Materials and Methods

### 2.1. Study Design

To answer the relevant question, a scoping review protocol was drafted using the Preferred Reporting Items for Systematic Reviews and Meta-Analyses Extension for Scoping Reviews (PRISMA-ScR) [13], which was revised and reviewed by all components of the research team. The final protocol was registered prospectively with the Open Science Framework on 29 April 2025 (https://osf.io/m7b8n/overview). Appendix A provides the PRISMA-ScR Checklist of the present study (Table A1).

### 2.2. Research Question

The review question was as follows: “Can stroke patients with very small or no ischemic brain lesions be safely submitted to early (within 6 days) extracranial carotid revascularization after systemic thrombolysis?” The question was developed using the PICO (population, intervention, comparator, and outcomes) framework.

### 2.3. Relevant Studies Identification

The aim of this study was to identify publications describing the results of extracranial carotid revascularization (either carotid endarterectomy—CEA—or carotid artery stenting—CAS) after systemic thrombolysis, which also provided timing of treatment in relation to thrombolysis and extension of the ischemic lesion at imaging.

Data collection included type of publication, study population, outcome data (including perioperative stroke or death, ICH, and neck hematoma), timing of treatment after thrombolysis and brain lesions dimension. When provided, the severity of stroke at admission, evaluated either with the National Institute of Health Stroke Scale (NIHSS) or with the modified Rankin scale (mRs), was also included to act as a surrogate marker of the extension of the ischemic lesion [11,12]. All results were reported qualitatively and compared to the results of delayed revascularization (i.e., >6 days) obtained by previous metanalysis on the topic. Evaluation of methodological quality of the studies was not performed, as it is not required by the scoping review guidelines.

Only English peer-reviewed journal papers published between 2005 and 2025 were included. Type of publications were randomized controlled trials, non-randomized controlled trials, observational studies (prospective and retrospective cohort studies), case–control studies, case series, individual case reports, and descriptive cross-sectional studies, as well as qualitative studies, systematic reviews, and meta-analyses that met the inclusion criteria.

Expert opinions and letters to the editor covering the topic of the review were also screened to identify eventual relevant data.

Three online databases (EMBASE, PubMed, and Cochrane CENTRAL) were used. A dedicated search on Google Scholar was also performed to identify potential studies that may not have been included in the selected databases (such as congress abstracts, communications at meetings, other). Studies predominantly centered on the use of mechanical thrombectomy or the treatment of tandem lesions (i.e., intracranial and extracranial internal carotid artery) were excluded, as they would act as confounding factors. Studies reporting only on the results in patients with extracranial internal carotid artery occlusion were also excluded. Reference lists of the selected studies were screened for possible additional data.

The full electronic search strategy for the EMBASE database is provided in Appendix B. The search strategies for the other databases were derived from this one.

### 2.4. Studies Selection

A software based on artificial intelligence (Rayyan, Qatar Computing Research Institute, Doha, Qatar.) [14] was utilized to identify duplicates. Exclusion of duplicates was then manually confirmed (no automated exclusion). Two reviewers (G.C., L.F.M.) independently evaluated all articles by title and abstract. Full texts of the articles that met the inclusion criteria were obtained, and a second review was conducted to confirm inclusion based on the full text. Any disagreements were resolved through discussion and consultation with a third reviewer (M.D.P.).

### 2.5. Data Charting Process

A data extraction table on Microsoft Word (Microsoft Corp, Redmond, WA, USA) was specifically developed to collect the relevant data for this review. The extraction variables (columns) included the following: author and journal of publication (if any), country of origin, year of publication, methodology/methods, population and sample size, intervention type (CAS or CEA), number of patients treated within 6 days, dimension of brain lesions/no lesions, severity of stroke (NIHSS/mRS), and outcomes of treatment (death, ischemic stroke, symptomatic intracranial hemorrhage(sICH)/asymptomatic intracranial bleeding (aICB), neck hematoma/bleeding requiring reoperation). Finally, a comment column was incorporated to provide a concise narrative synthesis of the principal findings of each study, offering a more detailed examination of the reported complications and an assessment of the respective strengths and limitations of each manuscript. The data extraction table was collaboratively developed by two reviewers (G.C., L.F.M.) to specify the variables for extraction. Each reviewer independently performed data charting. The results of data charting were subsequently discussed for each included paper by both reviewers to refine the instrument through an iterative process of discussion and revision and to ensure consistency and homogeneity of the extracted data. The same procedure of discussion/revision was applied when compiling the comment column of the data extraction table. A specific analysis of the outcomes was conducted whenever patient-level data were available within the article.

### 2.6. Reporting the Results

Data were reported in textual format as counts (*n*) and percentages (%), as means with standard deviations (SD), or as medians with ranges or interquartile ranges (IQR), as appropriate. Missing values were denoted by a dash (–). Incidence rates for predefined outcomes, together with corresponding 95% confidence intervals (CI), were calculated when sufficient data were available. Likewise, odds ratios (OR) were computed for outcomes with adequate data. Statistical significance was assessed using Fisher’s exact test, with a two-sided significance level set at *p* < 0.05. The analyses were performed using R (v4.5.2) (R Foundation for Statistical Computing, Vienna, Austria).

The comment column of the data extraction table was employed to correlate, when feasible, complications—particularly ICH—with their timing in relation to IVT administration and the degree of neurological impairment of each patient.

## 3. Results

### 3.1. Search Results and Selection of the Studies

Through the selected databases (PubMed, EMBASE, and Cochrane CENTRAL), a total of 872 publications were found. Four additional abstracts were added through research on Google Scholar and one through citation searches. After removing duplicates (*n* = 446), a total of 426 publications were manually screened using titles and abstracts.

Based on titles and abstracts, 384 publications were further removed, and the full-text versions of additional 2 articles could not be retrieved, thus leaving 40 articles to be evaluated through full text.

Of the 40 articles assessed, 24 were excluded for the following reasons: absence of data on lesion extension and/or stroke severity at admission (*n* = 4); lack of information regarding treatment timing after IVT (*n* = 5); and failure to include patients treated within 6 days of IVT or inability to isolate outcomes for this subgroup, as results were reported only in aggregate for the entire study population (*n* = 10).

Systematic reviews that did not provide additional primary data (*n* = 2) and meta-analyses (*n* = 3) were also excluded from the analysis. However, their findings were incorporated as comparative references in the discussion section.

Of the five studies identified through alternative search methods, three were excluded due to the absence of precise information regarding the timing of treatment, and one was excluded for failing to report data on patients treated within six days.

Overall, 17 studies were included in the present scoping review.

**Figure 1:**
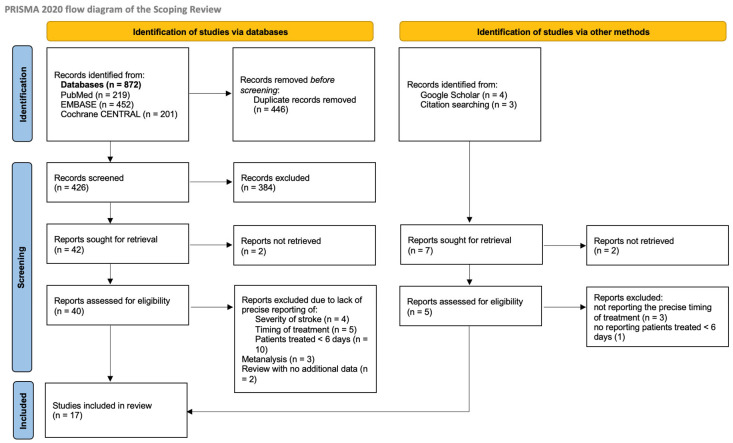
PRISMA flow diagram.

The detailed PRISMA-ScR flow diagram of study selection is shown in Figure 1.

### 3.2. Study Characteristics

Table 1 provides an overview of the characteristics of the included studies.

The evidence consisted of one case report [15] and multiple retrospective analyses: 14 single-center cohort studies [8,16,17,18,19,20,21,22,23,24,25,26,27], one two-center cohort review [28], and two analyses based on national databases [29,30]. No randomized controlled trials addressing this topic were identified through the literature search. Furthermore, nine studies incorporated a control arm, which included either patients with symptomatic carotid stenosis undergoing carotid revascularization without intravenous thrombolysis (*n* = 8) [16,18,19,22,23,29,30,31] or patients treated exclusively with intravenous thrombolysis without subsequent carotid revascularization (*n* = 1) [21].

Except for the two studies based on national databases, all cohorts were derived from university or tertiary care hospitals. Geographically, ten studies were conducted within the European Region (Italy: *n* = 3; France: *n* = 2; UK: *n* = 2; Sweden: *n* = 1; Finland: *n* = 1; Czech Republic: *n* = 1), four studies originated from the USA, and three from East Asia (Japan: *n* = 2; Republic of Korea: *n* = 1).

Three studies [8,16,27] specifically included patients who had previously/simultaneously undergone intracranial catheter-based interventions for the presenting stroke. All other studies did not address this specific issue in their Methods section; therefore, it was assumed this subset of patients was not included.

Although treatment of tandem lesions was an exclusion criterion for the present review, two of these studies [8,27] were already incorporated in the most comprehensive meta-analysis on the subject by Kakkos et al. [5]. The third one [16] was added following consensus among the reviewers (G.C., L.F.M., M.D.P.), as it employed a propensity score-matched design comparing patients undergoing revascularization without IVT, and the rate of intracranial catheter-based interventions was relatively low (15/56, 26.7%). Moreover, this cohort displayed a contemporary approach to stroke management, supporting its relevance.

Patient-level data were available exclusively for small series.

**Table 1:** Characteristics of included studies (*n* = 17). IVT: intravenous thrombolysis; CEA: carotid endarterectomy; CAS: carotid artery stenting.

	Author Journal	Year	Country	Design of the Study and Duration	Population and Sample Size	Intervention Type
1	Bellomo T. et al. [16] Annals of Vascular Surgery	2024	USA	Retrospective analysis of a single-center cohort (Mass. General Hospital). 2007–2019	56 stroke pts submitted to carotid revascularization after IVT (4.9%) CONTROL: 1083 symptomatic pts with no IVT submitted to carotid revascularization (94.1%)	35 CEA, 21 CAS (15 catheter-based interventions for stroke: 9 before CEA and 6 before CAS)
2	Doo Hyuk Kwon et al. [17] Journal of Korean Medical Sciences	2022	Republic of Korea	Retrospective analysis of a cohort of emergent carotid intervention at a single center (Keimyung University School of Medicine). 2005–2020	19 pts with stroke submitted to emergent CAS after IVT (11 ICA occlusions excluded) No CONTROL Group	19 CAS: 11 pts with ICA occlusion, 6 pts with tandem lesion (not considered in the review), 2 pts with ICA stenosis (included in the scoping review)
3	Johal A.S. et al. [29] European Journal of Vascular and Endovascular Surgery	2021	UK	Retrospective study using a large population-based dataset from the National Vascular Registry in the United Kingdom (UK-NVR). 2014–2019	1055 stroke pts submitted to IVT and CEA (11.7%) CONTROL: 7975 stroke pts with no IVT submitted to CEA (88.3%)	CEA (patients with CAS or carotid bypass were excluded from the study)
4	Deiana G. et al. [28] Annals of Vascular Surgery	2020	Italy	Retrospective analysis of a cohort of patients from two centers (Brotzu Hospital, Cagliari, Italy and Santissima Annunziata Hospital, Sassari, Italy). 2016–2018	11 stroke pts submitted to CEA after IVT (15.7%) CONTROL: 59 symptomatic pts with no IVT submitted to CEA (84.3%)	CEA under locoregional anesthesia in all patients
5	Ijäs P. et al. [8] Stroke	2018	Finland	Retrospective analysis of a single-center cohort of patients (Helsinki University Hospital, Helsinki, Finland). Comparison between pts operated <48 h after IVT and pts operated >48 h and <14 days. 2005–2016	128 pts submitted to CEA after IVT (15.7%) CONTROL: 777 stroke pts with no IVT submitted to CEA (84.3%)	All CEA, no data regarding surgical technique or anesthesia
6	Gunka I. et al. [18] Annals of Vascular Surgery	2017	Czech Republic	Retrospective analysis of a single-center cohort of patients (Charles University Hospital, Prague, Czech Repuplic). 2013–2016	13 pts submitted to CEA after IVT (14%)—4 patients with acute extracranial carotid occlusion CONTROL: 80 symptomatic pts with no IVT submitted to CEA (86%)	CEA under locoregional anesthesia (general anesthesia in selected patients)
7	Adachi K. et al. [19] Neurosurgical Review	2017	Japan	Retrospective analysis of a single-center cohort of patients treated with CAS < 2 wks of stroke (Fujita Health University, Japan). 2009–2014	16 patients undergoing urgent/emergent CAS for stroke/stroke in evolution—4 receiving IVT (25%) No CONTROL	CAS with embolic protection
8	Yamamoto Y. et al. [15] The Journal of Medical Investigation	2016	Japan	Case report on emergent CEA after IVT for deteriorating symptoms (Tokushima Prefectural Miyoshi Hospital, Japan).	One emergent CEA due to fluctuating symptoms	CEA under general anesthesia
9	Azzini C. et al. [20] European Journal of Vascular and Endovascular Surgery	2016	Italy	Retrospective analysis of a single-center cohort of patients treated with CEA (also <12 h) after IVT (Azienda Ospedaliera-Universitaria, Ferrara, Italy). 2009–2014	34 stroke pts submitted to CEA after IVT CONTROL: IVT pts without CEA	CEA under general anesthesia with routine shunt
10	Bazan H.A. et al. [21] Journal of Vascular Surgery	2015	USA	Retrospective analysis of a single-center cohort of patients treated with CEA/CAS after IVT (Ochsner Clinic, New Orleans, Louisiana). 2009–2015	31 stroke pts submitted to carotid revascularization after IVT CONTROL: 134 no IVT pts submitted to CEA (33% TIA pts)	IVT: CEA 25 − CAS 6 No IVT: CEA 110 − CAS 24 CEA under general anesthesia using standard patch angioplasty with routine intraoperative shunt
11	Vellimana A.K. et al. [22] Neurosurgery	2014	USA	Retrospective analysis of a single-center cohort of patients treated with CEA after IVT (Washington University School of Medicine, St. Louis, Missouri). 1995–2007	11 stroke pts submitted to carotid revascularization after IVT CONTROL: 131 no-IVT pts submitted to CEA (symptomatic—TIA included but percentage not specified)	CEA under general anesthesia. Intraoperative neuromonitoring with EEG. Selective shunting on EEG indication
12	Koraen-Smith L. et al. [30] Stroke	2014	Sweden	Retrospective analysis of a prospectively collected national database (Swedvasc and Riks-Stroke). 2008–2012	79 stroke pts submitted to carotid revascularization after IVT CONTROL: 3919 no IVT pts submitted to CEA (symptomatic stenosis)	IVT: CEA 71 − CAS 6 + 2 pts with aborted TEA due to high bifurcation No IVT: CEA 110 − CAS 24
13	Yong Y.P. et al. [23] Journal of Vascular Surgery	2013	UK	Retrospective analysis of a single-center cohort of patients treated with CEA after IVT (Nottingham University Hospitals, Nottingham, UK). 2010–2012	Seven stroke pts submitted to CEA after IVT No CONTROL	CEA under locoregional or general anesthesia
14	Sallustio F. et al. [24] Stroke Research and Treatment	2012	Italy	Retrospective analysis of a single-center cohort of patients treated with CAS after IVT (Policlinico Tor Vergata, Rome, Italy). 2006–2011	Six stroke pts submitted to CAS after IVT No CONTROL	CAS under local anesthesia with embolic protection device (distal filter)
15	Leseche G. et al. [25] Journal of Vascular	2012	France	Retrospective analysis of a single-center cohort of patients with STROKE IN EVOLUTION (Bichat-Claude Bernard University Hospital, Paris, France). 2003–2010	Seven pts with stroke in evolution (fluctuating symptoms) after IVT No CONTROL	CEA under locoregional anesthesia/general anesthesia with routine shunting
16	Bartoli M.A. et al. [26] European Journal of Vascular and Endovascular Surgery	2008	France	Retrospective analysis of a single-center cohort of patients treated with CEA after IVT (Hopital de la Timone, Service de Chirurgie Vasculaire, Marseille, France). 2005–2008	12 stroke pts submitted to CEA after IVT No CONTROL	IVT: CEA 12 CEA under general anesthesia (technique at surgeon’s discretion)
17	Abou-Chebl A. et al. [27] Stroke	2005	USA	Retrospective analysis of a single-center cohort of patients treated with CAS after IVT + GPIIb/IIIa Antagonists (Cleveland Clinic, Cleveland, Ohio). (Time period not specified)	12 stroke pts submitted to emergent endovascular treatment and IVT + GPIIb/IIIa Antagonists (2 patients receiving CAS) No CONTROL	CAS in two patients associated with endovascular treatment of intracranial lesion

### 3.3. Results

#### 3.3.1. Severity of Stroke at Admission and Timing Between IVT and Carotid Revascularization

The results of the included studies are reported in Table 2.

A total of 1459 patients were included across 17 studies. Of these, 35 patients (2.4%) underwent CAS, while 1424 patients (97.6%) received CEA.

All the included studies reported results for patient treated within 2 weeks from IVT. Concerning the timing variable addressed in the scoping review—specifically, treatment within six days following intravenous thrombolysis (IVT)—data for 402 patients were identified. However, several studies reported timing in narrower intervals (seven studies < 24 h [15,17,18,19,20,27], two studies < 48 h [8,20], and four studies < 72 h [8,21,28,30]) rather than providing aggregated data for the <6-day timeframe.

With respect to the second component of the scoping review question—namely, the extent of the ischemic brain lesion—eight studies [17,18,19,20,21,24,25,26] reported this parameter as an exclusion criterion, meaning that carotid revascularization was performed exclusively in patients whose brain lesions were below this predefined size. Specifically, four studies [21,24,25,26] included patients with lesions involving less than one-third of the middle cerebral artery (MCA) territory, one study [20] applied an Alberta Stroke Program Early CT Score (ASPECTS) ≥ 8, and three studies [17,18,19] employed both criteria.

One study provided a brief qualitative description of the lesions [23], while another also reported the mean ischemic lesion volume for the entire cohort [24]. Only a single case report described treatment in a patient with an ischemic lesion exceeding one-third of the MCA territory [15].

Conversely, seven studies [8,16,22,27,28,29,30] did not provide any information regarding lesion size.

The severity of stroke at admission was instead well-reported in all studies. Three studies reported the median value of the modified Rankin scale (mRs) [16,28,29], while the other 14 used the National Institute of Health Stroke Scale (NIHSS) [15,17,18,19,20,21,22,23,24,25,26,27,30].

Notably, all studies reporting the mRS included a statistically significant higher proportion of patients with disabling stroke (mRS ≥ 3) in the IVT group compared to the control group (patients with no IVT), with prevalence ranging from 18.2% to 57% [16,28,29].

Studies reporting NIHSS scores predominantly included patients with moderate stroke (NIHSS 5–15). NIHSS values were presented either as median with range (Ijäs et al.: 6(0–20); Gunka et al.: 7 (3–18); Yong et al.: 15 (10–22)) [8,18,23] or as mean with standard deviation (overall mean: 10.2 ± 2.8) [17,21,22,23,25,26,27,31]. Patient-level NIHSS data were reported in nine studies [15,17,18,19,22,23,24,26,27]. Across these studies, individuals undergoing CAS generally exhibited higher baseline NIHSS scores, with an overall mean of 13.4 ± 1.5.

#### 3.3.2. Ischemic Stroke at 30 Days

One study (Johal et al., 1055 patients) did not differentiate outcomes for ischemic versus hemorrhagic stroke, whereas the series by Vellimana et al. (11 patients) reported only the incidence of hemorrhagic stroke [22,29]. After excluding these two cohorts, the 30-day incidence of ischemic stroke was 10 events among 393 patients (2.5%; 95% confidence interval [CI], 1.2–4.6).

Among these ten events, one major stroke resulting in death occurred in a patient who underwent surgery within 24 h of intravenous thrombolysis (IVT) and presented with unstable neurological symptoms [18]. Additionally, one ischemic stroke was reported in a patient operated within 48 h [8], one retinal infarction in a patient treated at 3 days, and one minor stroke at 5 days post-IVT [30]. Four strokes occurred in patients operated after 48 h, although the exact timing was not clearly specified [8], and for two patients, the timing of postoperative stroke was not reported [16].

No detailed information regarding lesion extent or severity of stroke at admission was available for patients who experienced postoperative ischemic stroke.

#### 3.3.3. Asymptomatic Intracranial Bleeding (aICB) Plus Symptomatic Intracranial Hemorrhage (sICH) at 30 Days

Johal et al. did not distinguish outcomes between ischemic and hemorrhagic stroke [29]. After excluding these cohort, the 30-day incidence of aICB plus sICH was 16 events among 397 patients (4.0%; 95% CI, 2.1–6.0). When restricting the analysis to symptomatic cases only, the incidence decreased to 10 of 397 patients (2.5%; 95% CI, 1.0–4.1), as 6 patients exhibited radiological findings without clinical symptoms. Notably, 13 of 16 events (81.2%) occurred within 48–72 h following IVT.

Regarding the type of revascularization, sICH occurred in three patients in the CAS group (3/35, 8.6%; 95% CI, 0–17.9) compared with seven patients in the CEA group (7/362, 1.9%; 95% CI, 0.5–3.4).

Patients undergoing CAS demonstrated significantly higher odds of developing sICH compared with those undergoing CEA (OR = 4.75; 95% CI, 1.17–19.28; *p* = 0.049).

The precise extent of brain lesions in patients with sICH or aICB could not be determined from the analyzed studies. Patient-level data on stroke severity were available for 10 individuals (9 with NIHSS scores and 1 with mRS), comprising three cases of aICB and seven cases of sICH, drawn from six studies [19,21,22,23,26,28]. Among patients with aICB, one-third (33.3%) presented with a modified Rankin Scale (mRS) score of 5 at admission, indicative of a disabling stroke, while the remaining two had mRS scores between 0 and 2. All patients with sICH exhibited moderate stroke severity according to NIHSS (range: 6–23), with 5 of 7 (71.4%) presenting with NIHSS scores greater than 10 at admission.

#### 3.3.4. Overall Stroke (Ischemic and Hemorrhagic) at 30 Days

For the entire cohort included in the scoping review, the combined incidence of ischemic stroke and hemorrhagic complications was 45 out of 1459 cases (3.1%; 95% CI, 2.2–4.0). This composite outcome encompassed ischemic stroke, sICH, and aICB.

No detailed information regarding lesion extent or severity of stroke at admission was available for patients who experienced postoperative ischemic stroke.

#### 3.3.5. Death at 30 Days

Similarly, excluding the subset of patients from Vellimana et al. [22], the rate of death at 30 days for the remaining studies was instead 17/1484 (1.1%; 95% CI, 0.6–1.7).

#### 3.3.6. Wound Bleeding/Access Site Complications at 30 Days

No series including CAS patients reported access site complications (bleeding/pseudoaneurysm). Excluding the subset of patient from Vellimana et al., whose study only reported the outcome of sICH [22], the rate of wound bleeding/access site complication at 30 days was 55/1484 (3.7%; 95% CI, 2.7–4.7).

Only two studies specifically focused on patients requiring reoperation [8,30], whereas three studies also included cases of neck hematoma or wound complications that did not necessitate surgical reintervention [16,21,29]. The temporal distribution of wound complications was assessed exclusively by Johal et al., who reported a statistically significant increase in wound complication rates when CEA was performed within five days following IVT [29].

## 4. Discussion

Patients requiring extracranial carotid revascularization following IVT represent a small subset of the overall stroke population treated with IVT; however, their proportion has progressively increased over the past two decades, currently accounting for approximately 10% of all CEA for symptomatic patients [29].

Although the primary research question of this scoping review could not be definitively addressed due to limitations in the available evidence, several key observations emerged:

1. Low risk of ischemic stroke and mortality after CEA: The incidence of postoperative ischemic stroke and death among patients undergoing CEA after IVT appears to be low.
2. Higher complication risk with CAS, particularly sICH: Patients treated with CAS exhibit a higher risk of complications, notably sICH. However, this results should be interpreted with caution for several reasons. First, it is important to recognize that this cohort represents a distinct clinical subset, as CAS is frequently performed in the hyperacute phase (within hours) in patients with severe strokes and is often combined with intracranial thrombectomy for tandem lesions. These patients typically present with more severe neurological deficits and are exposed to the additional hemorrhagic risk of dual antiplatelet therapy [17,27], although the combination of IVT and dual antiplatelet therapy does not appear to be associated with an increased incidence of sICH, as suggested by recent findings from Cavalcante et al. [31]. Second, it must be emphasized that there is a marked numerical imbalance between the CEA (*n* = 1424) and CAS (*n* = 35) cohorts: this substantial volume disparity and inherent selection bias further limits any possible assumption.
3. Wound complications after CEA: The reported incidence of wound-related complications following CEA after IVT is higher, largely influenced by the retrospective analysis by Johal et al. involving 1055 IVT patients [29]. Notably, this outcome encompassed bleeding and hematomas not necessarily requiring surgical reintervention, and the exact number of reoperations was not specified, leaving uncertainty regarding the clinical significance of these events.
4. Limited data on infarct size as a selection criterion: The extent of cerebral infarction after IVT is rarely analyzed in the context of carotid revascularization and is primarily used as an exclusion criterion (typically, patients are considered for intervention if the infarct size is less than one-third of the middle cerebral artery territory and/or the ASPECT Score is ≥8). Although infarct size is a recognized risk factor for sICH [10], none of the patients who developed sICH had documented infarct dimensions in the reviewed studies. This limited characterization of the extent of brain lesions represents an intrinsic limitation of all available studies and substantially hampers the identification of potential risk factors that could aid in patient selection.
5. Stroke severity as a determinant of complications: Stroke severity at admission was more consistently reported, and NIHSS scores were available for most patients who experienced sICH. In this subgroup, over 70% had NIHSS > 10, indicating moderate stroke severity; one patient had a modified Rankin Scale score of 5, reflecting a disabling stroke. Stroke severity is increasingly acknowledged as a major determinant of perioperative complications in the setting of carotid revascularization after stroke [4,32].
6. Timing of revascularization and risk of sICH: Most postoperative sICH events occurred when revascularization was performed within 48–72 h of IVT, while the remaining occurred more than two weeks after IVT. No cases were observed between 4 and 6 days post-IVT in our analysis. The pooled sICH rate among patients undergoing CEA was 1.9%, comparable to rates reported in stroke patients undergoing CEA without prior IVT [10,33].

Symptomatic intracranial hemorrhage remains the most critical and feared complication following carotid revascularization in patients who have received IVT. The current body of evidence is inconclusive: while most studies report no increased risk of sICH after CEA in this setting [8,9,16,18,21,25,28,29,30], others (two studies, both coming from the same authors) suggest a higher incidence of intracranial bleeding [22,34]. A recent metanalysis on the association of IVT to acute stenting (and its required antiplatelet therapy) also underlines that the risk of sICH might be lower than previously feared in the specific setting of tandem lesions [31], thus further supporting a more proactive approach to carotid revascularization.

The Society for Vascular Surgery guidelines recommend delaying carotid revascularization for at least 48 h in clinically stable stroke patients (without previous IVT) to mitigate the risk of sICH and other neurological complications [35], as corroborated by the metanalysis of Hasan et al. [36]. Although not specifically targeted to the evaluation of IVT patients, this study further supports the higher risk of early (<48 h) revascularization of stroke patients also reported by our scoping review. More recently, two meta-analyses have proposed an even longer delay—approximately 6 to 7 days—before intervention for the subset of patients with previous IVT [5,37]. Although such postponement may reduce hemorrhagic risk, it must be weighed against the substantial risk of recurrent ischemic stroke, which can reach 8–22% within the first week [3].

This recommendation has been incorporated into the latest European Society for Vascular Surgery guidelines, which state the following: “For patients with acute ischemic stroke due to a symptomatic 50–99% carotid stenosis who have received IVT, delaying CEA or CAS by six days following completion of thrombolysis should be considered (Class IIa, Level of Evidence B).” However, these same guidelines acknowledge that postponing revascularization in patients without ischemic lesions at post-IVT brain imaging could be unnecessary [3].

The evidence underpinning this recommendation primarily derives from one of the aforementioned meta-analyses [5] and a single large retrospective study by Vellimana et al., based on the National Inpatient Sample (NIS) [34].

A critical appraisal of these sources is warranted before generalizing their conclusions to all IVT-treated patients, particularly given the rarity of sICH. Notably, both meta-analyses included asymptomatic intracranial hemorrhages in their outcome measures, as exemplified by the study by Deiana et al. [28], who reported three such events detected exclusively through routine postoperative imaging, which was systematically performed in all IVT patients regardless of neurological status. This approach may have led to underreporting of hemorrhages in the non-IVT group, where imaging was performed only in cases of neurological deterioration. An additional case of sICH reported by Fortin et al. [38] occurred in a patient with a large hemispheric infarction who underwent surgery 15 days after IVT. Given the prolonged interval, the causal relationship with IVT appears doubtful, although the event was counted in both metanalysis. Interestingly, Kakkos et al. [5] conducted a sensitivity analysis excluding studies with delayed (>7 days) or unreported intervention timing, and this yielded a nonsignificant odds ratio for intracranial bleeding for CEA after IVT versus CEA with no IVT (OR 1.66; 95% CI 0.17–16.43).

Furthermore, the dominant influence of the NIS-based study on pooled estimates introduces significant limitations. While its large sample size (551 IVT patients and 120,738 non-IVT patients) is a strength, the NIS is an administrative rather than a clinical database, whose possible inaccuracy in the setting of carotid revascularization has already been reported [39]. Moreover, the inclusion of transient ischemic attacks (without a defined percentage) in the control group compromises the reliability of comparative risk estimates.

Overall, the available studies on carotid revascularization following IVT consist exclusively of retrospective analyses derived from single-center or multicenter registries, resulting in substantial heterogeneity regarding outcome definitions, inclusion criteria, and timing of revascularization. The absence of prospective investigations and randomized controlled trials significantly limits the strength of the conclusions that can be drawn from the current evidence.

Findings from this scoping review are not conclusive and underscore the need for prospective studies to clarify optimal timing and patient selection for carotid revascularization after IVT, particularly regarding the characteristics of brain lesions. Multicenter registries are needed due to the low number of adverse events.

## 5. Conclusions

Further multicenter prospective registries addressing carotid revascularization after IVT are warranted to refine management strategies for this specific patient population, in order to avoid excessive delays. Future studies should clearly define outcome measures and complication criteria prior to enrollment, including the incidence of recurrent ischemic events during the waiting period for CEA or CAS, the role of medical treatment with single or dual antiplatelet therapy after IVT, and a thorough characterization of brain lesions at admission and after IVT, as these aspects have been poorly or not addressed in the available literature. Although a randomized controlled trial would be ethically acceptable and scientifically desirable, its feasibility appears limited given the low frequency of adverse events reported in the current literature.

## Figures and Tables

**Table 2:** Results of the included studies. ASPECT: Albert stroke program early CT score; NIHSS: National Institute of Health Stroke Scale; mRs: modified Rankin score; IVT: intravenous thrombolysis; MCA: middle cerebral artery; ICH: intracranial hemorrhage; ECASS HI: European Cooperative Acute Stroke Study Hemorrhagic Infarction; IQR: interquartile range; CEA: carotid endarterectomy; CAS: carotid artery stenting; AMI: acute myocardial infarction.

	Author Journal	*n* of Patients Treated Within 6 Days	Dimension of Brain Lesion/ASPECT Score	NIHSS/mRs *n* (%)	Overall Timing of Treatment	Outcome	Comment
1	Bellomo T. et al. [16] Annals of Vascular Surgery	48/56 (85.7%)	–	mRs 0:8 (14%) mRs 1:6 (11%) mRs 2:5 (9%) mRs 3:16 (29%) mRs 4:16 (29%) mRs 5:5 (9%)	IVT: Median 3 days (0–25) No IVT: 20 pts at day 0 (1.9%)	Ischemic stroke: 4% (2/56) ICH/Bleeding: 0% Wound bleeding/Reop.: 2% (1/56) Death: 5% (3/56) (statistically higher at univariate but not at multivariate)	Propensity score matching to no IVT patients. No difference in outcome between revascularization with IVT and without IVT (stroke, death, bleeding).
2	Doo Hyuk Kwon et al. [17] Journal of Korean Medical Sciences	All	ASPECTS ≥ 8 in all patients (<1/3 MCA in all patients)	NIHSS mean +/− SD: 15.1 +/− 6.4	Emergent (<4 h from symptom)	Ischemic stroke: 0% ICH/Bleeding: 2/8 (25%, all asymptomatic ECASS HI Type 2) Death: 1/8 (12.5%, cardiac arrest) Access bleeding: 0%	Patients treated in an emergent setting. Very high NIHSS at admission. The two patients with no tandem lesion had no neurologic complication but one died of cardiac arrest at 3 days. Very long study time with small numbers.
3	Johal A.S. et al. [29] European Journal of Vascular and Endovascular Surgery	342/1055 (32.4%) of which 199 (18.9%) between day 0 and 5	–	mRS ≥ 3 (IVT 23.5% vs. no IVT 16.9%, *p* < 0.001). mRs 0:139 (13.2%) mRs 1:357 (33.8%) mRs 2:311 (29.5%) mRs 3:196 (18.6%) mRs 4:49 (4.6%) mRs 5:3 (0.3%)	IVT: Median + IQR 10 days (6–17) No IVT: Median + IQR 11 days (7–20)	Stroke at 30 days (ischemic and hemorrhagic): IVT 19/1055 (1.8%) vs. no IVT 170/7975 (2.1%) Death at 30 days: IVT 9/1055 (0.9%) vs. no IVT 53/7975 (0.7%) Neck hematoma: IVT 39/1055 (3.7%) vs. no IVT 182/7975 (2.3%) *p* < 0.05 For patients between 0 and 5 days: Stroke = IVT 2.0% vs. no IVT 2.3% Neck hematoma = IVT 4.5% vs. no IVT 2.0% *p* < 0.05	No statistically different rate of stroke and death IVT vs. no IVT. Statistically significant higher rate of neck hematoma in patients treated < 5 days. The registry does not differentiate between postoperative hemorrhagic or ischemic stroke. Neck hematoma was a clinical condition; not all patients required reoperation.
4	Deiana G. et al. [28] Annals of Vascular Surgery	At least three but not specifically defined—all IVT patients treated within 14 days	–	mRS after IVT: 0–2 in 9 patients (81.8%) 3–5 in 2 patients (18.2%) mRs in no IVT: 0–2 in 54 patients (91.5%) 3–5 in 5 patients (8.5%)	IVT: Median + range 8 days (2–13) No IVT: Median + range 26 days (1–175)	Death at 30 days: IVT 0% vs. no IVT 0% Ischemic Stroke at 30 days: IVT 0% vs. no IVT 1.7% (1/59) ICH at 30 days: IVT 3/11 (27.2%) vs. no IVT 3.4% (2/59) Neck hematoma: –	Control group including all symptomatic patients (i.e., also TIA and amaurosis fugax). All patients with ICH were treated within 3 days from IVT. All ICH were radiologic findings with no clinical relevance
5	Ijäs P. et al. [8] Stroke	7 pts (5.5%) < 24 h 20 pts (15.6%) < 48 h 28 pts (21.9%) < 72 h 87 pts (68%) < 2 wks	–	NIHSS Median + Range: 6 (0–20)	IVT: Median 9 days (Range: 0–349)	The rate of any ICH related to IVT (from hemorrhagic infarction to parenchymal hemorrhage) was 3.9%. Death at 90 days: IVT 0% vs. no IVT 0% Ischemic Stroke at 30 days: 3.9% (5/128) ICH/hemorrhagic transformation: 2.3% (3/128)—1 operated < 48 h, 1 at 19 days, 1 at 54 days Exploration because of neck hematoma: 8.6% (11/128)	Stroke recurrence in IVT patients was 5.5% at median 4 days after IVT (range, 0–8 days). Time between IVT and CEA was not associated with CEA-related complications. High rate of stroke recurrence during the waiting time for CEA. No clear definition of ICH.
6	Gunka I. et al. [18] Annals of Vascular Surgery	Four pts with ICA occlusion and failed IVT: immediate CEA (median 117.5 min) Two pts with unstable neurological status with CEA < 24 h Seven pts with stable neurological status with CEA < 2 wks	ASPECTS ≥ 8 in all patients (<1/3 MCA in all patients)	NIHSS median + range: 7 (3–18)	IVT: Median + Range 2 days (0–13) No IVT: Median + Range 6 days (0–14)	Stroke/Death at 30 days: IVT 7.7% (1/13) vs. no IVT 5% (4/80) Symptomatic ICH: IVT 0% vs. no IVT 1.2% (1/80) Exploration because of neck hematoma: IVT 0% vs. no IVT 3.8% (3/80)	Cohort of patients including four ICA occlusion. No difference between CEA + IVT and pts with CEA + no IVT.
7	Adachi K. et al. [19] Neurosurgical Review	Three out of four (75%) patients—two pts < 24 h, one pts < 24 h after symptom deteriotration, one pts atat 14 days	ASPECTS ≥ 8 in all patients (<1/3 MCA in all patients)	NIHSS: 7, 9, 14, 23	All patients treated within 2 wks	Symptomatic ICH: 50% (2/4)	Severe ICH (one requiring decompression). These two pts all treated < 24 h, with the highest NIHSS (14, 23).
8	Yamamoto Y. et al. [15] The Journal of Medical Investigation	10.5 h after IVT (atat admission ICA occlusion—recanalization after IVT)	Extensive lesion at MRI (>1/3 MCA territory)	NIHSS 14	Single emergent CEA case	No ICH, no other complications	Amelioration of symptoms. Postoperative NIHSS 5.
9	Azzini C. et al. [20] European Journal of Vascular and Endovascular Surgery	22 pts treated < 48 h: 11 emergent CEA < 12 h 11 pts with CEA > 12 h and <48 h	Emergent pts: ASPECT Score 10 in eight pts, 9 in two, 8 in one Evidence of a significant salvageable ischemic penumbra on perfusion CT	Emergent pts: mean NIHSS 10.0 +/− 6.3 (range 6.0–23.0) CEA > 12 h and <48 h: mean NIHSS 13.2 +/− 8.6 CEA > 48 h and <2 wks: mean NIHSS 7.0 +/− 2.5	All 34 pts treated within 2 wks 11 emergent CEA < 12 h 11 pts with CEA > 12 h and <48 h 12 pts with CEA > 48 h and within 2 wks	Emergent pts: stroke 0%, ICH 0%, death-AMI 1/11 (9%) 11 pts with CEA > 12 h and <48 h: ICH/hemorrhagic transformation: 18% (2/11)—no ischemic stroke, no death 12 pts with CEA > 48 h and within 2 wks: ICH/hemorrhagic transformation: 8.3% (1/12)—no ischemic stroke, no death	No major complications, except one death due to AMI. Three cases of hemorrhagic transformation, all asymptomatic, described as petechial (two cases) and hematoma (one case).
10	Bazan H.A. et al. [21] Journal of Vascular Surgery	17/31 (54.8%) pts treated < 72 h 14/31 pts 44.2%) treated > 72 h and <7 days	<1/3 of infarct volume on CT or MRI of the head (Infarct detected at MRI 80% of patients)	IVT mean NIHSS 6.6 (range 0–19) No IVT mean NIHSS 6.1 (range 0–26)	IVT: mean 2.2 days (range 0–7) No IVT: mean 2.2 days (range 0–15)	Death at 30 days: IVT 6.5% (2/31) vs. no IVT 1.5% (2/134) Ischemic Stroke at 30 days: IVT 0% vs. no IVT 0.7% (1/134) Symptomatic ICH: 3.2% (1/31) vs. 1.5% (2/134) Neck hematoma (not specified if requiring reoperation): IVT 3.2% (1/31) vs. 1.5% (2/134)	The stroke + death + AMI rates at 30 day: IVT 9.7% (3/31) vs. no IVT 4.5% (6/134—*p* = 0.38). No differences in individual complications between the two groups. 26% of pts in the IVT group with acute carotid occlusion. All ICH were symptomatic. The ICH in IVT group was a CAS < 72 h for ICA occlusion with NIHSS 15 No ICHs in pts with NIHSS score of <10 (both IVT and no IVT).
11	Vellimana A.K. et al. [22] Neurosurgery	IVT: 4/11 pts Not specified in the no IVT group	–	IVT mean NIHSS 10.2 +/− 8.7 (range 0–24) No IVT: –	IVT: mean 38.3 +/− 53.9 days (range 1–181) No IVT: –	Death at 30 days: – Ischemic Stroke at 30 days: n.a. Symptomatic ICH: IVT 18.2% (2/11) vs. no IVT 0.8% (1/131) Neck hematoma (not specified if requiring reoperation): n.a.	Very long study time with relatively low numbers (consecutive series). IVT as the only risk factor for ICH at multivariate analysis; however, the type of patients in no IVT is poorly described. Only study with negative results—all patients with ICH treated < 72 h.
12	Koraen-Smith L. et al. [30] Stroke	8 within 72 h (58 within 2 weeks—38 within 10 days)—Patient-level data not available	–	IVT mean NIHSS 8 (range 2–25) No IVT mean NIHSS: n.a.	IVT: median 10 days (Range 0–108) No IVT: median 9 days (range 0–178)	Death at 30 days: IVT 0% vs. no IVT 31/3626 (0.9%) Ischemic stroke at 30 days: IVT 2/79 (2.5%) vs. no IVT 62/3626 (1.7%) Symptomatic ICH: 0% vs. 27/3626 (0.7%) Neck hematoma (requiring reoperation): IVT 3/79 (3.8%) vs. 119/3626 (3.3%)	No statistically significant difference between IVT and no IVT. The two strokes in the IVT were both minor. No correlation between postoperative complications and time from lysis to intervention. All complications in IVT occurring within 1 week (presenting symptom: four minor strokes, one major stroke)
13	Yong Y.P. et al. [23] Journal of Vascular Surgery	Four patients within 7 days	5 small subacute infarction, 1 none, 1 small subacute with petechial hemorrhage	IVT median NIHSS 15 (range 10–22)	IVT: median 7 days (Range 2–12)	Death at 30 days: 0% Ischemic stroke at 30 days: 0% Symptomatic ICH: 1/7 (14.2%) Neck hematoma (requiring reoperation): 0%	One symptomatic ICH in one patient with NIHSS 10 operated at 48 h from IVT (hypertension at the end of CEA). No other complications.
14	Sallustio F. et al. [24] Stroke Research and Treatment	All patients treated within 7 days	<1/3 MCA in all patients Median DWI Lesions volume 26 cm3 (range 6–52)	Mean NIHSS 12 (IQR 9–16)	Median 48 h (IQR 30–94)	Death at 30 days: 0% Ischemic stroke at 30 days: 0% Symptomatic ICH: 0% Access site complication: 0%	Only study giving the extension of the lesion. Stents patent at 12 months. One recurrent stroke due to new onset atrial fibrillation
15	Leseche G. et al. [25] Journal of Vascular	3/7(42.8%) pts < 6 days	<1/3 MCA in all patients	Mean NIHSS 9 (range 5–15)	Median 6 days (range 2–13)	Death at 30 days: 0% Ischemic Stroke at 30 days: 0% Symptomatic ICH: 0% Neck hematoma: 0%	Total of 7 pts in a group of 27 with stroke in evolution. The entire group had only one AMI as complication.
16	Bartoli M.A. et al. [26] European Journal of Vascular and Endovascular Surgery	5/12 (41.7%) pts treated < 6 days	<1/3 of infarct volume on CT or MRI of the head	IVT mean NIHSS 12 (range 5–21)	IVT: median 8 days (range 1–16)	Death at 30 days: 0% Ischemic stroke at 30 days: 0% Symptomatic ICH: 1/12 (8.3%) Neck hematoma (not specified if requiring reoperation): 0%	ICH patient operated at 33 h from onset of symptoms due to floating thrombus and tight ICA stenosis (present also intracranial ICA/MCA occlusion). ICH after the patient became hypertensive. No other complications.
17	Abou-Chebl A. et al. [27] Stroke	1 h, 3 h	–	NIHSS 16, 17	All < 10 h	Death at 30 days: 2/12 (16.6%) Ischemic Stroke at 30 days: 0% ICH: 2/12 16.36%) Bleeding complications: 0%	In the subgroup treated with CAS for extracranial disease, there was no ICH and no asymptomatic bleeding.

## Data Availability

No new data were created/presented.

## Appendix A

Preferred Reporting Items for Systematic reviews and Meta-Analyses extension for Scoping Reviews (PRISMA-ScR) Checklist.

**Table A1:** PRISMA-ScR Checklist. # number of the corresponding page.

Section	Item	PRISMA-ScR Checklist Item	Reported on Page #
**TITLE**			
Title	1	Identify the report as a scoping review.	Page 1
**ABSTRACT**			
Structured summary	2	Provide a structured summary that includes (as applicable): background, objectives, eligibility criteria, sources of evidence, charting methods, results, and conclusions that relate to the review questions and objectives.	Page 1
**INTRODUCTION**			
Rationale	3	Describe the rationale for the review in the context of what is already known. Explain why the review questions/objectives lend themselves to a scoping review approach.	Page 2
Objectives	4	Provide an explicit statement of the questions and objectives being addressed with reference to their key elements (e.g., population or participants, concepts, and context) or other relevant key elements used to conceptualize the review questions and/or objectives.	Page 2
**METHODS**			
Protocol and registration	5	Indicate whether a review protocol exists; state if and where it can be accessed (e.g., a Web address); and if available, provide registration information, including the registration number.	Page 3
Eligibility criteria	6	Specify characteristics of the sources of evidence used as eligibility criteria (e.g., years considered, language, and publication status) and provide a rationale.	Page 3
Information sources	7	Describe all information sources in the search (e.g., databases with dates of coverage and contact with authors to identify additional sources), as well as the date the most recent search was executed.	Page 3
Search	8	Present the full electronic search strategy for at least one database, including any limits used, such that it could be repeated.	Appendix B
Selection of sources of evidence†	9	State the process for selecting sources of evidence (i.e., screening and eligibility) included in the scoping review.	Page 4
Data charting process	10	Describe the methods of charting data from the included sources of evidence (e.g., calibrated forms or forms that have been tested by the team before their use, and whether data charting was performed independently or in duplicate) and any processes for obtaining and confirming data from investigators.	Page 4
Data items	11	List and define all variables for which data were sought and any assumptions and simplifications made.	Page 4
Critical appraisal of individual sources of evidence	12	If performed, provide a rationale for conducting a critical appraisal of included sources of evidence; describe the methods used and how this information was used in any data synthesis (if appropriate).	Not appropriate
Synthesis of results	13	Describe the methods of handling and summarizing the data that were charted.	Page 4
**RESULTS**			
Selection of sources of evidence	14	Give numbers of sources of evidence screened, assessed for eligibility, and included in the review, with reasons for exclusions at each stage, ideally using a flow diagram.	Figure 1
Characteristics of sources of evidence	15	For each source of evidence, present characteristics for which data were charted and provide the citations.	Table 2
Critical appraisal within sources of evidence	16	If performed, present data on critical appraisal of included sources of evidence (see item 12).	Not appropriate
Results of individual sources of evidence	17	For each included source of evidence, present the relevant data that were charted that relate to the review questions and objectives.	Page 19–20, Table 2
Synthesis of results	18	Summarize and/or present the charting results as they relate to the review questions and objectives.	Page 19–20
**DISCUSSION**			
Summary of evidence	19	Summarize the main results (including an overview of concepts, themes, and types of evidence available), link to the review questions and objectives, and consider the relevance to key groups.	Page 21
Limitations	20	Discuss the limitations of the scoping review process.	Page 22
Conclusions	21	Provide a general interpretation of the results with respect to the review questions and objectives, as well as potential implications and/or next steps.	Page 23
**FUNDING**			
Funding	22	Describe sources of funding for the included sources of evidence, as well as sources of funding for the scoping review. Describe the role of the funders of the scoping review.	Not applicable

## Appendix B

EMBASE Search Strategy (Literature Search performed: 25 April 2015).

SEARCH QUERY, “(‘carotid artery stenting’/exp OR ‘carotid artery stenting’ OR ‘carotid endarterectomy’/exp OR ‘carotid endarterectomy’) AND (‘stroke’/exp OR ‘stroke’) AND (‘thrombolysis’/exp OR ‘thrombolysis’) NOT (‘mechanical thrombectomy’/exp OR ‘mechanical thrombectomy’) NOT (‘tandem’/exp OR ‘tandem’) AND [english]/lim AND [2005–2025]/py”.
